# Ribosome-dependent conformational flexibility changes and RNA dynamics of IRES domains revealed by differential SHAPE

**DOI:** 10.1038/s41598-018-23845-x

**Published:** 2018-04-03

**Authors:** Gloria Lozano, Rosario Francisco-Velilla, Encarnacion Martinez-Salas

**Affiliations:** Centro de Biología Molecular Severo Ochoa, Consejo Superior de Investigaciones Científicas - Universidad Autónoma de Madrid, Nicolás Cabrera 1, 28049 Madrid, Spain

## Abstract

Internal ribosome entry site (IRES) elements are RNA regions that recruit the translation machinery internally. Here we investigated the conformational changes and RNA dynamics of a picornavirus IRES upon incubation with distinct ribosomal fractions. Differential SHAPE analysis of the free RNA showed that nucleotides reaching the final conformation on long timescales were placed at domains 4 and 5, while candidates for long-range interactions were located in domain 3. Salt-washed ribosomes induced a fast RNA local flexibility modification of domains 2 and 3, while ribosome-associated factors changed domains 4 and 5. Consistent with this, modeling of the three-dimensional RNA structure indicated that incubation of the IRES with native ribosomes induced a local rearrangement of the apical region of domain 3, and a reorientation of domains 4 and 5. Furthermore, specific motifs within domains 2 and 3 showed a decreased flexibility upon incubation with ribosomal subunits *in vitro*, and presence of the IRES enhanced mRNA association to the ribosomal subunits in whole cell lysates. The finding that RNA modules can provide direct IRES-ribosome interaction suggests that linking these motifs to additional sequences able to recruit trans-acting factors could be useful to design synthetic IRESs with novel activities.

## Introduction

The function of RNA molecules depends on their three-dimensional (3D) structure^1^ and also on their ability to acquire distinct conformations on its own and/or in response to specific signals^2^. Conformational transitions could be spatially and temporally tuned to achieve distinct functions enabling complex ribonucleoproteins (RNPs) to be assembled in a hierarchical ordered manner. In addition, RNA conformational changes occurring over broad timescales can range from local rearrangements in motifs involved in tertiary interactions to large global remodeling in the orientation of helices^3^.

Nucleotides involved in tertiary interactions may have unusual backbone or stacking geometries or undergo conformational changes on different timescales^4,5^. As such, distinct RNA local nucleotide dynamics can be detected by computing the differential reactivity obtained using slow- and fast-reacting selective 2′-hydroxyl acylation analyzed by primer extension (SHAPE) reagents. For instance, isatoic anhydride (IA) is a slow reagent (430 s half life at 37 °C) while 1-methyl-6-nitroisatoic (1M6) is a fast reagent (31 s half life at 37 °C). Nucleotides with enhanced reactivity toward IA are usually in the rare C2′-endo conformation, experience slow local dynamics, and in some cases, govern the folding of entire RNA domains^4^. 1M6 is an IA derivative that stacks with RNA nucleobases with one face available for stacking, which occurs in long-range interactions, bulges, turns, or at the termini of some helices^4^. On the other hand, 1-methyl-7-nitroisatoic (1M7) is a fast-reacting compound^6^ that, unlike 1M6, does not stack with nucleobases^7^. The RNA reactivity toward these compounds provides information on nucleotides that undergo local conformational changes on long timescales (IA) and those involved in tertiary interactions (1M6)^5,8^, hence time-dependent RNA-ligand interactions.

Translation control is a key step in gene expression regulation in all organisms. In eukaryotes, the vast majority of mRNAs initiates translation by a cap-dependent mechanism that depends on the recognition of the m^7^G(5′)ppp(5′)N structure (designated cap) placed at the 5′ end of most mRNAs^9^. This process begins with the binding of the translation initiation factor (eIF)-4F complex to the cap. This complex recruits the 40S ribosomal subunit bound to eIF3, eIF2, and the initiator tRNA, and scans the 5′UTR until an AUG triplet is found in the appropriate context to start protein synthesis. Joining of the 60S ribosomal subunit follows this step, producing a translation competent complex. However, specific mRNA regions referred to as internal ribosome entry sites (IRES), can recruit the 40S ribosomal subunit through a cap-independent mechanism (reviewed in^10^).

RNA structure determines the function of viral IRES elements^11^. However, different IRES elements perform the same function despite lacking conservation of primary sequence, secondary RNA structure, and host factor requirement to recruit the ribosomal subunits^12^. Internal initiation of translation can occur either by interaction of the IRES with the 40S ribosomal subunit, or by binding to initiation factors, which mediate the recruitment of the 40S ribosomal subunit. For instance, the dicistrovirus intergenic region (IGR) or the hepatitis C (HCV) IRES physically associate the 40S subunit *in vitro*^13,14^. Nevertheless, there are notable differences among these IRESs. Whereas the IGR assembles a complex with 80S ribosomes in the absence of eIFs^15,16^, the HCV IRES, and also those of picornaviruses, require different combinations of eIFs to assemble 48S complexes *in vitro*^17,18^.

The picornavirus IRES elements are prototypes for understanding the mechanism of ribosome recruitment due to their requirement of factors for translation initiation. Previous structural analyses have shown that the IRES of encephalomyocarditis virus (EMCV) and foot-and-mouth disease virus (FMDV) are arranged in structural domains (designated H to L, or 2 to 5, respectively)^11^. Although RNA probing data revealed a stable secondary structure of the FMDV IRES, the nucleotides involved in the dynamic folding and in tertiary interactions of this regulatory element remain unknown. Moreover, although it is well established that domains 4 and 5 provide the binding site for initiation factors, the specific role of domains 2 and 3 is less well understood, despite containing binding sites for proteins such as PTB or Ebp1. Indeed, it was hypothesized that domains 2 and 3 could contribute to promote the specific IRES conformation critical for the interaction with the translation machinery^19^.

Here we have investigated the RNA conformational flexibility and RNA dynamics of the FMDV IRES by differential SHAPE in the presence of various components of the cellular translation machinery. We show that the ribosomal subunits induced fast structural changes on domains 2 and 3, whereas host factors mostly induced slow structural changes on domains 4 and 5. Consistent with our results, analysis of the mRNA association to ribosomal subunits inside cells indicated that an IRES-containing mRNA remained bound to both subunits more prominently than an mRNA lacking the IRES.

## Results

### Native ribosomes modify the conformational flexibility of IRES domains

**Figure 1:**
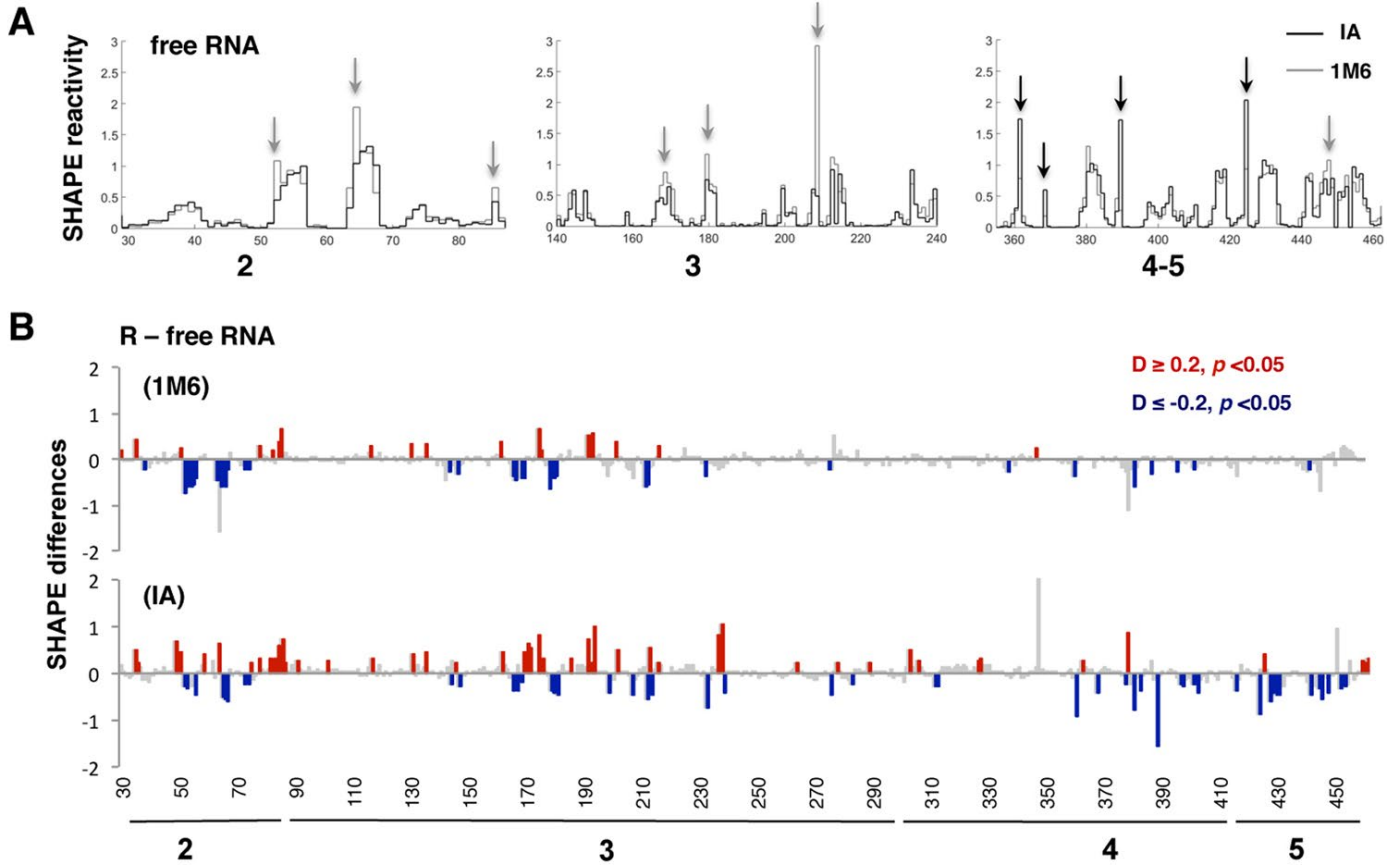
Conformational changes in the RNA local flexibility of the IRES incubated with native ribosomes. (**A**) Reactivity patterns for IA (black) and 1M6 (gray) reagents obtained after QuSHAPE software processing for representative regions of domain 2, 3 and 4–5 of the free IRES element. Black and grey arrows depict some examples of enhanced reactivity toward IA or 1M6, respectively. (**B**) Statistically significant SHAPE differences towards IA and 1M6 of the RNA incubated with R fraction relative to free RNA as a function of the nucleotide position. Red or blue bars depict nucleotides with *p*-values < 0.05 and absolute SHAPE reactivity differences higher or lower than 0.2, respectively. Grey bars depict non-statistically significant differences (*p*-values > 0.05 and/or absolute differences D <0.2).

IRES elements promote initiation of translation by recruiting the ribosome internally. However, even within the picornavirus IRESs, there are different strategies to recruit the translation machinery involving unique roles for the individual IRES domains. Aiming at understanding the role of RNA structural domains on ribosome recruitment, we studied the local flexibility of the FMDV IRES element incubated with ribosomal fractions. To gain information about the conformational changes of the IRES, we took advantage of differential SHAPE methodology using reagents that provide information on slow and fast conformational changes of the RNA structure^4^. Hence, the slow reagent IA provided information on nucleotides that undergo local conformational changes on long timescales^5^, and the fast reagent 1M6 informed of nucleobases that achieve their conformation on short timescales^4^. The RNA local flexibility of the free IRES was first analyzed using the normalized reactivity (mean ± SD) towards IA (n = 9) and 1M6 (n = 5) (Supplementary Fig. S1A, Dataset 1, Dataset 2). Representation of the normalized reactivity pattern of the free IRES revealed marked differences toward IA (black arrows) or 1M6 (grey arrows) in specific positions of domains 2, 3, 4, and 5 (Fig. 1A). The reactive positions are located in loops of the secondary structure (Supplementary Fig. S1B).

**Figure 2:**
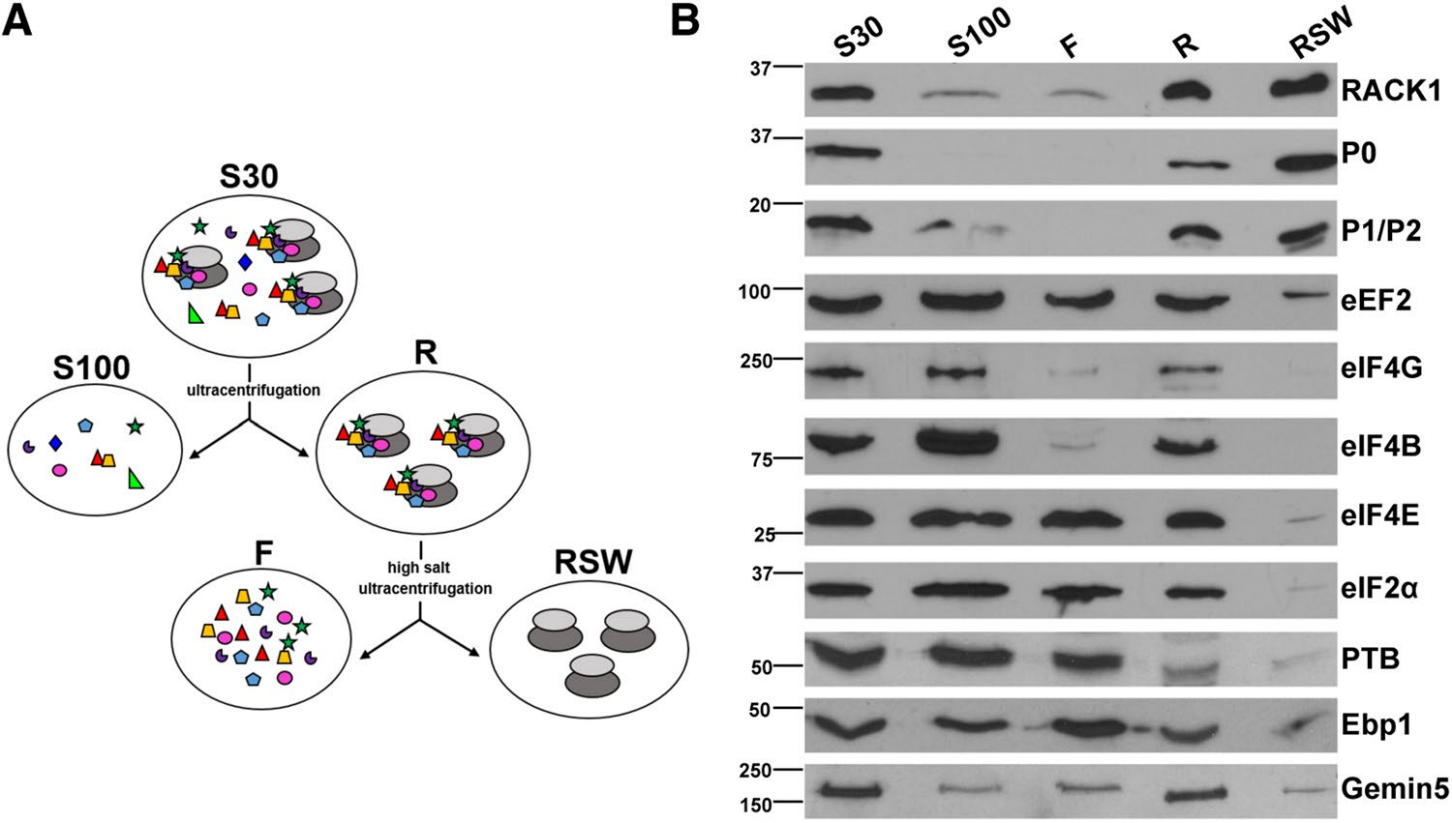
Preparation of cellular fractions. (**A**) Schematic of the procedure used to prepare cellular fractions S30, S100, R, RSW, and F from HEK293 cells. S30 extract is the total lysate obtained from cells. S30 ultracentrifugation yielded the S100 fraction (supernatant), and the ribosomes plus associated factors (R) (pellet). To prepare the fraction containing ribosomes free from associated factors (RSW), the ribosomal pellet was dissolved in high-salt buffer, loaded in a discontinuous sucrose gradient and ultracentrifuged. The supernatant of the ultracentrifugation yielded the F fraction. (**B**) HEK293 fractions corresponding to S30, S100, F (100 µg of total protein), ribosomes (R) and salt-washed ribosomes (RSW) (30 µg) were analyzed by Western blot on the same membrane to detect the presence of RACK1 (40 S subunit), the 60S ribosomal proteins P0 and P1/P2, the elongation factor eEF2, the initiation factors eIF4G, eIF4B, eIF4E, and eIF2α, and the IRES-interacting proteins PTB, Ebp1 and Gemin5. This figure shows horizontal slices of the WB carried out for each factor. Images of the un-cropped WB film obtained for each factor are shown in Supplementary Fig. S7).

Next, we prepared cellular fractions with distinct content of ribosomes (Fig. 2A). Briefly, ultracentrifugation of the cytoplasmic lysate (S30) rendered the soluble fraction free of ribosomes (S100), and a pellet consisting of ribosomes with associated cellular factors (R). Then, high-salt washing of the pellet provided the salt-washed ribosome (RSW) fraction, which contains ribosomes without (or with little) associated factors (F). The relative amount of diagnostic proteins present in these fractions was analyzed by immunoblotting (Fig. 2B). The 40S ribosomal protein RACK1 was detected in all fractions, as expected^20^. Conversely, the 60S ribosomal proteins P0 and P1/P2 were immunodetected in the fractions containing ribosomes (S30, R, and RSW)^21^. The elongation factor eEF2 was present in all fractions, although it was significantly reduced in RSW. Furthermore, the initiation factors eIF2α, eIF4B, eIF4G, and eIF4E were detected in S30, S100, F, and R fractions to different extents, but not in RSW, showing that the high salt washing effectively removed factors from the ribosomes. In addition, we evaluated the presence of the IRES-interacting factors PTB, Ebp1 and Gemin5 in the cellular fractions. The three proteins were detected in S30, S100, F, and R fractions with different intensity. Similarly to the analyzed eIFs, these factors were strongly reduced in RSW.

Next, to evaluate the impact of the ribosomal fractions on the IRES conformational flexibility, we performed differential SHAPE analysis in the presence of the soluble fraction S100 and the native ribosome extract (R), using IA (slow reagent) and 1M6 (fast reagent). The effect on the local RNA flexibility was determined computing the SHAPE difference, subtracting the normalized reactivity obtained for the free RNA to that obtained for the IRES incubated with the different fractions. In all cases, data represent the mean ± SD from at least 3 independent experiments, and only the statistically significant SHAPE differences (*p* < 0.05), and absolute values D > 0.2 were taken into consideration. The differences of SHAPE reactivity with free RNA rendered positive values (more accessible residues), and negative values (less accessible residues). In this study we focused on the nt positions that decrease their reactivity, which could be the result of either a more constrained RNA structure or protein/ribosome interaction. Conversely, analysis of nt with increased reactivity would render positions with more relaxed conformation. The results indicated that incubation of the IRES transcript with S100 did not produce significant differences relative to the free RNA with any of these compounds, IA or 1M6 (Supplementary Fig. S2). These results suggest that the concentration of the RNA-binding proteins interacting with the IRES region present in the supernatant was not sufficient to induce a reorganization of the IRES structure. In contrast, the SHAPE difference profile obtained for the IRES region incubated with native ribosomes (R) relative to free RNA showed statistically significant changes in domains 2 and 3 with both, 1M6 and IA (Fig. 1B). Interestingly, domains 4 and 5 exhibited significant differences of reactivity mostly with IA. These results indicate that the conformational changes in domains 2 and 3 of the IRES incubated with the R fraction occur at short timescales, whereas changes in domains 4 and 5 require long timescales to achieve their final conformation.

**Figure 3:**
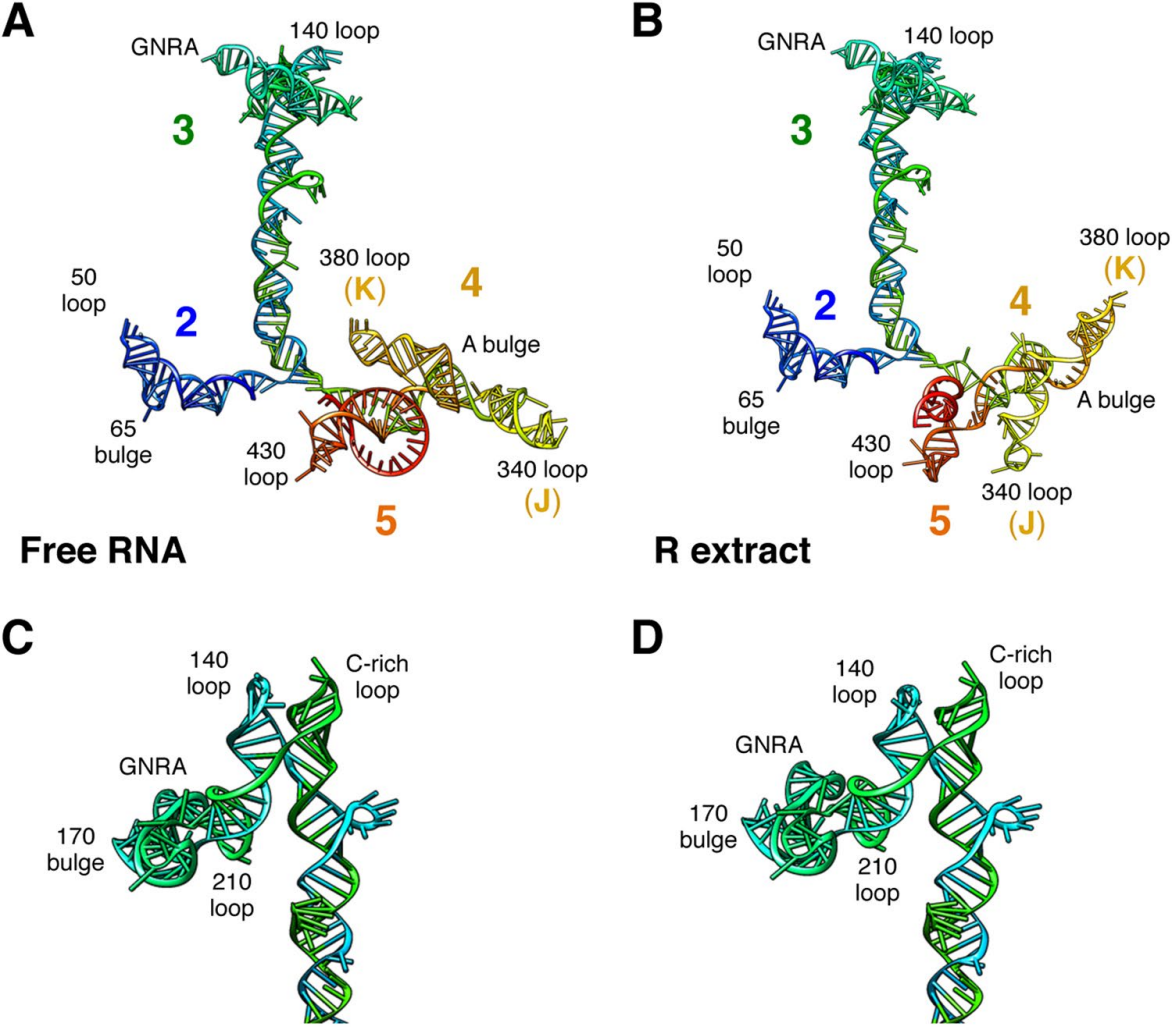
RNA structure modeling of the IRES region. Predicted 3D structure models for the IRES imposing SHAPE reactivity values obtained for the free RNA (**A**) and upon incubation of the IRES transcript with the R extract (**B**). Domains 2, 3, 4, and 5, subdomains J and K of domain 4, as well as the GNRA tetraloop, loops, and bulges referred to as in the text are indicated. Detailed structure model of the apical region of domain 3 for the free RNA (**C**), and incubated with R extract (**D**).

To gain information about the IRES conformational changes induced by the interaction with R fraction we took advantage of the RNAstructure software incorporating the SHAPE reactivity data, and the RNAComposer server. The predicted structure of the free IRES element treated with IA is depicted in Fig. 3A. Upon incubation with R extract no major changes in the 3D structure of domain 2 were visualized (Fig. 3B). However, modification of 3D structure of the apical region of domain 3 resulted in a local rearrangement of residues belonging to the GNRA tetraloop and the 140 loop, that, together with the C-rich loop, act as a hinge (Fig. 3C,D). Moreover, the conformational changes of domains 4 and 5 observed with IA severely modified the 3D structure, changing the orientation of subdomains J and K, reorganizing the A bulge of domain 4, as well as the single-stranded region of domain 5 (compare Fig. 3A,B). Altogether, these results indicated that upon incubation of the IRES with the R fraction, domains 3, 4, and 5 undergo significant changes in their three-dimensional structure.

### Salt-washed ribosomes and ribosome-associated factors induce conformational changes on different domains of the IRES

**Figure 4:**
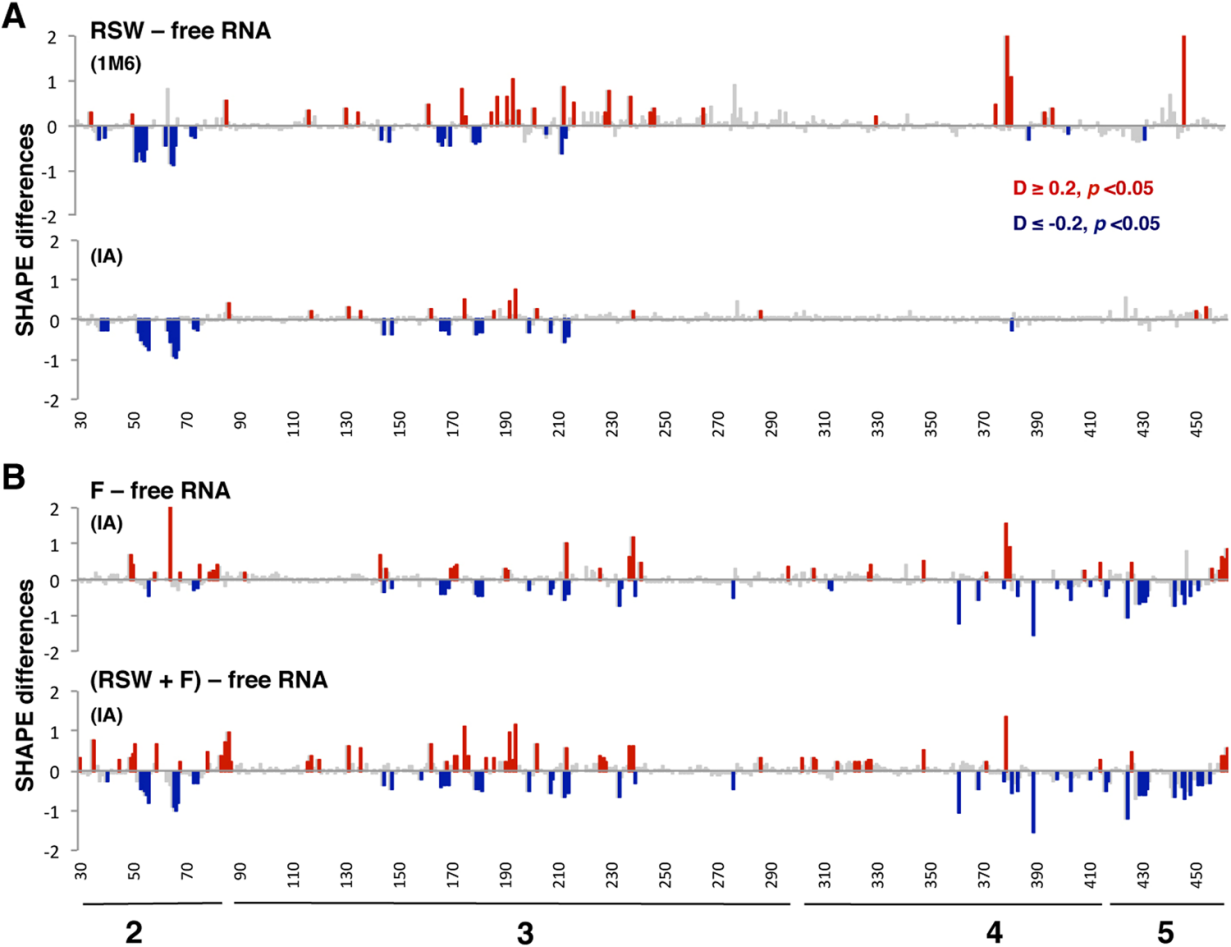
Modification of the RNA local flexibility of the IRES incubated with salt-washed ribosomes. (**A**) Statistically significant SHAPE differences towards 1M6 and IA reagents of the RNA incubated with RSW fraction relative to free RNA as a function of the nucleotide position. (**B**) Statistically significant SHAPE differences towards IA of the RNA incubated with F, or RSW + F fractions relative to free RNA as a function of the nucleotide position. Symbols as in Fig. 1B.

The above results indicated that native ribosomes promote structural changes within specific domains of the IRES in different timescales. We wondered whether ribosomes free of associated factors could perform similar IRES conformational flexibility changes to native ribosomes. To this end, we incubated the IRES transcript with the salt-washed ribosome fraction (RSW) (Fig. 2) prior treating samples with IA or 1M6 reagents. The results showed statistically significant differences relative to free RNA in domains 2 and 3 with both, 1M6 and IA (Fig. 4A). The values of SHAPE differences were similar for both compounds, and also similar with those observed in the presence of the R extract for 1M6 (Fig. 4A compared to Fig. 1B). However, no gross negative values were observed in domains 4 and 5 in the presence of RSW (Fig. 4A). Consistent with the lack of major differences in SHAPE reactivity, the 3D model of the IRES incubated with the RSW fraction largely resembles the free RNA (Supplementary Fig. S3).

These results prompted us to analyze if ribosome-associated factors could be responsible for the conformational changes observed on the domains 4 and 5 upon incubation with R extract (Fig. 1B). To test this possibility, we used the fraction designated F which contained a significant amount of IRES-interacting proteins as shown for PTB, Ebp1, and Gemin5 (Fig. 2). The difference profile relative to the free RNA revealed that the F fraction induced changes affecting domains 3, 4, and 5 (Fig. 4B), largely coincident with those observed with the R fraction (Fig. 1B), except for the reactivity decrease in domain 2. Then, we analyzed the IRES local flexibility in the presence of the RSW extract supplemented with the F fraction to determine if the reconstituted mix could reproduce the pattern obtained with native ribosomes (R). The difference profile revealed that combination of the fractions RSW and F restored the pattern observed in the presence of the R extract alone (Fig. 4B).

Collectively, these results illustrate unique structural features of the IRES element. First, the ribosome free of factors could induce modifications on the conformational flexibility of domains 2 and 3 of the IRES element (the 5´end domains). Second, the cellular factors associated to native ribosomes, as illustrated for eIF4G, eIF4B, PTB, Ebp1 or Gemin5, could be responsible for the changes in domains 4 and 5 (the 3´end domains).

### 40S and 60S ribosomal subunits induce structural changes within domains 2 and 3 of the IRES element

**Figure 5:**
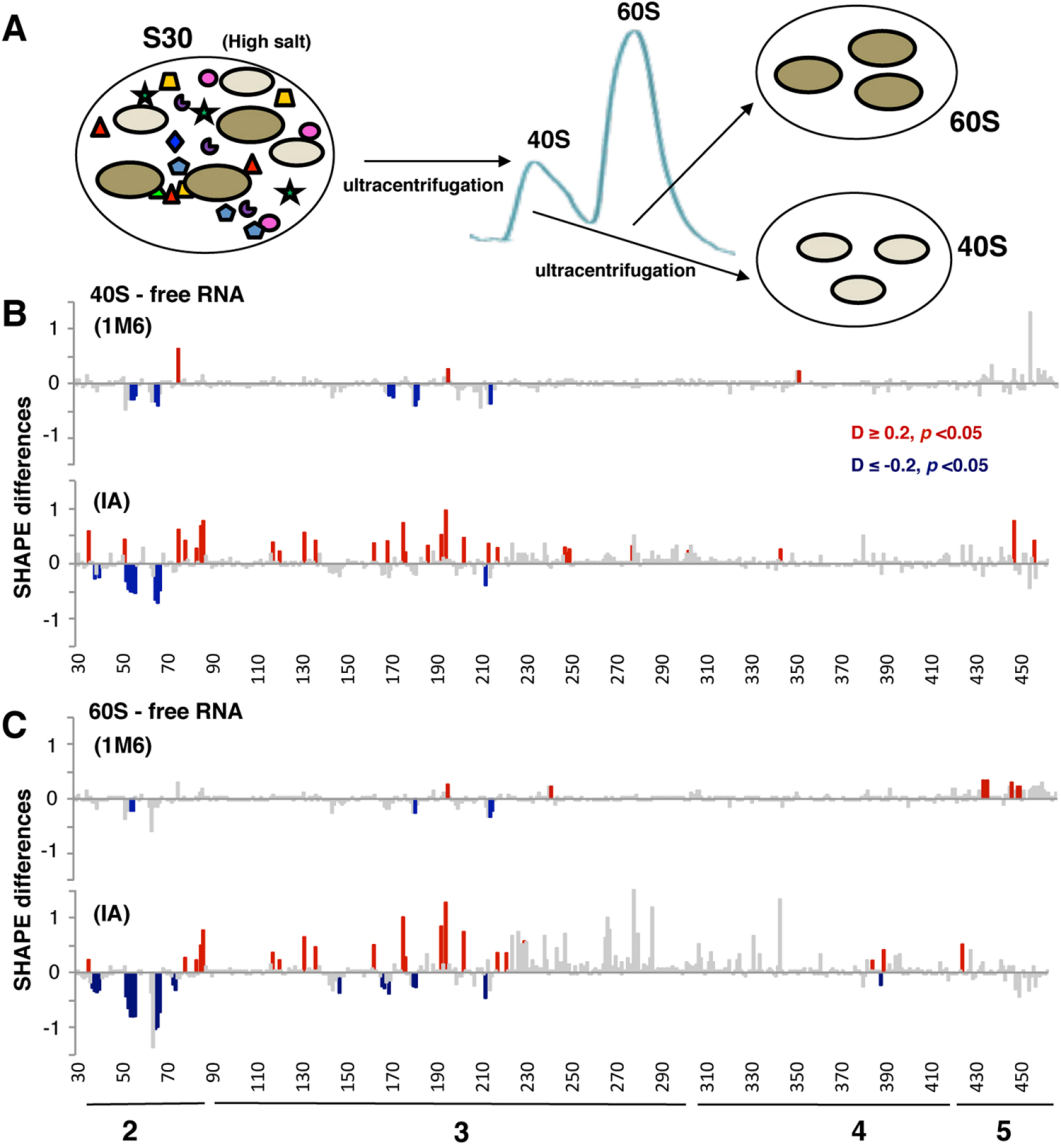
Conformational changes in the local flexibility of the IRES incubated with 40S or 60S ribosomal subunits. (**A**) Preparation of 40S and 60S ribosomal subunits from HEK293 cells. S30 extracts prepared with high salt buffer were loaded in continuous sucrose gradients and ultracentrifuged to separate the fractions corresponding to 40S and 60S peaks. These fractions were ultracentrifuged to obtain the purified ribosomal subunits. Significant SHAPE differences towards 1M6 and IA of the RNA incubated with 40S subunits (**B**) or 60S (**C**) relative to free RNA as a function of the nucleotide position. Symbols as in Fig. 1B.

The observation that the ribosome fraction free of factors (RSW) induced reactivity changes on the IRES altering the local flexibility of domains 2 and 3 (Fig. 4A) prompted us to analyze whether the individual ribosomal subunits could reproduce the pattern of IRES reactivity differences. Hence, we performed differential SHAPE assays of the IRES RNA in the presence of purified 40S and 60S subunits, which were prepared from dissociation ribosomal profiles of HEK293 cell lysates incubated with high salt to remove the associated factors (Fig. 5A)^22^.

The IA and 1M6 reactivity profiles (mean ± SD) obtained for the IRES RNA incubated with 40S and 60S are shown in Supplementary Fig. S4, Datasets 1 and 2. Relative to the free RNA, a significant decrease of SHAPE reactivity was observed in domain 2, being more intense for IA than 1M6 (Fig. 5B,C). Moreover, the IA difference profiles obtained in the presence of 40S or 60S subunits revealed statistically significant changes largely coincident with the RSW pattern (see Fig. 4A). We noticed that although the SHAPE difference induced by 40S and 60S were located on similar positions, the reactivity decrease observed in the presence of 60S subunits was slightly stronger. Furthermore, given that these changes were robustly detected with IA, and weakly with 1M6 (Fig. 5B,C), we conclude that they occur on long timescales.

### Presence of the IRES in mRNA enhances the association to the ribosomal subunits inside cells

**Figure 6:**
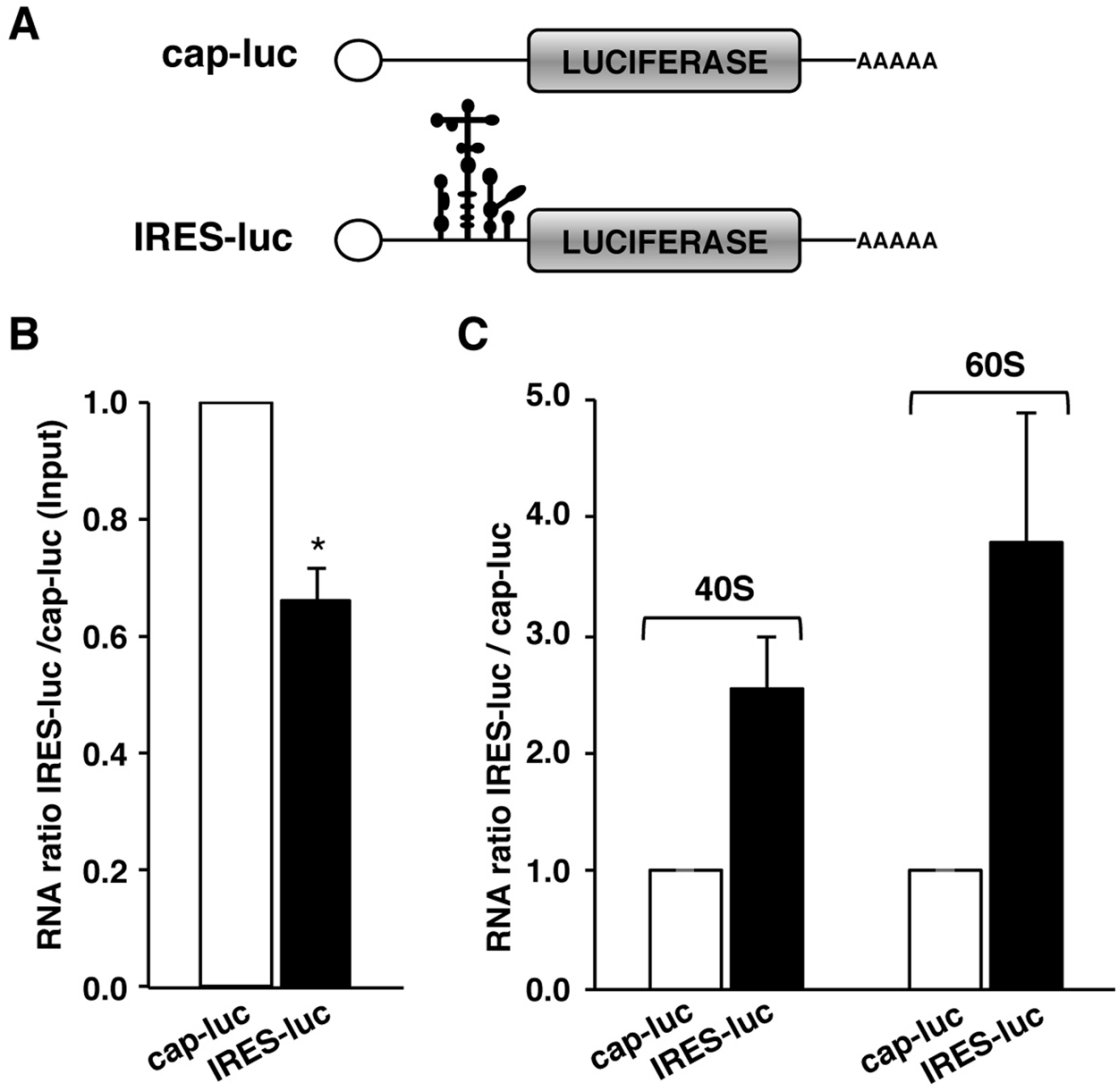
Association of IRES-RNA and cap-RNA to ribosomal subunits in whole cell lysates. (**A**) Schematic of transcripts cap-luc and IRES-luc. (**B**) Quantification of IRES-RNA levels (black bar) relative to cap-RNA (white bar) present in total cell lysates monitored by RT-qPCR. (**C**) Quantification of IRES-RNA relative to cap-RNA levels associated to 40S and 60S subunits. Values represent the mean ± SD obtained in two independent assays. Asterisks denote p-values (*p< 0.05).

Since the individual 40S and 60S ribosomal subunits induced conformational changes on similar positions of the IRES incubated *in vitro*, we decided to investigate these results using an independent approach. Hence, we studied if an mRNA that contained a functional IRES element could remain associated to the ribosomal subunits in the cellular context in the absence of associated-ribosome factors in comparison to a cap-mRNA. Thus, ribosomal subunit association was analyzed using two reporter transcripts that only differ in the presence of the IRES element (Fig. 6A). HEK293 cells were transfected with plasmids expressing the cap-luc or IRES-luc transcripts, and luciferase expression was used to monitor gene expression in transfected cells (Supplementary Fig. S5A). Next, to determine the relative amounts of cap-luc and IRES-luc RNAs in transfected cells, we analyzed the RNA levels in total cell lysates by RT-qPCR. The IRES-luc RNA accounted for 0.66 of the cap-luc mRNA (Fig. 6B). This difference in RNA levels was used to normalize the RNA copies associated to ribosomal particles.

To monitor the association of cap-luc and IRES-luc mRNAs to individual ribosomal subunits, we fractionated cell lysates prepared in high-salt buffer in dissociation gradients^22^. Following RNA extraction of the fractions corresponding exclusively to 40S and 60S peaks (see Fig. 5A), we analyzed the content of IRES-luc or cap-luc transcripts by RT-qPCR. The relative amount of the RNAs bound to each ribosomal subunit was calculated as the ratio of IRES-luc to cap-luc (set to 1) for the individual 40S and 60S subunits. The IRES-luc RNA was associated with both ribosomal subunits to a larger extent than the cap-luc RNA (2.6-fold in 40S, and 3.8-fold in 60S) (Fig. 6C). Moreover, comparison of the RNA bound to 60S relative to 40S subunits indicated that, although the amount of cap-luc and IRES-luc transcripts associated to 60S was very modest, the IRES-luc RNA levels were higher than those of cap-luc RNA (Supplementary Fig. S5B).

In summary, these data showed that the IRES-containing mRNA remained directly associated to both ribosomal subunits in whole cell extracts (mainly to the 40S subunit), and also that the IRES-containing mRNA remained bound to ribosomal subunits more efficiently than the cap-mRNA.

### Incubation of the IRES with ribosome fractions differentially modifies the IRES RNA dynamics and tertiary interactions

Although the secondary structure of the FMDV IRES has been reported^11^, the RNA dynamics of this regulatory element remain to be elucidated. In addition, since we found that ribosomes and ribosome-associated factors interact with different domains of the IRES in a time-dependent manner, we decided to analyze the RNA dynamics for the free IRES and the differences relative to its ligand-bound states (R and RSW). This was performed calculating IA-1M6 differences, as well as 1M6-1M7^4^.

**Figure 7:**
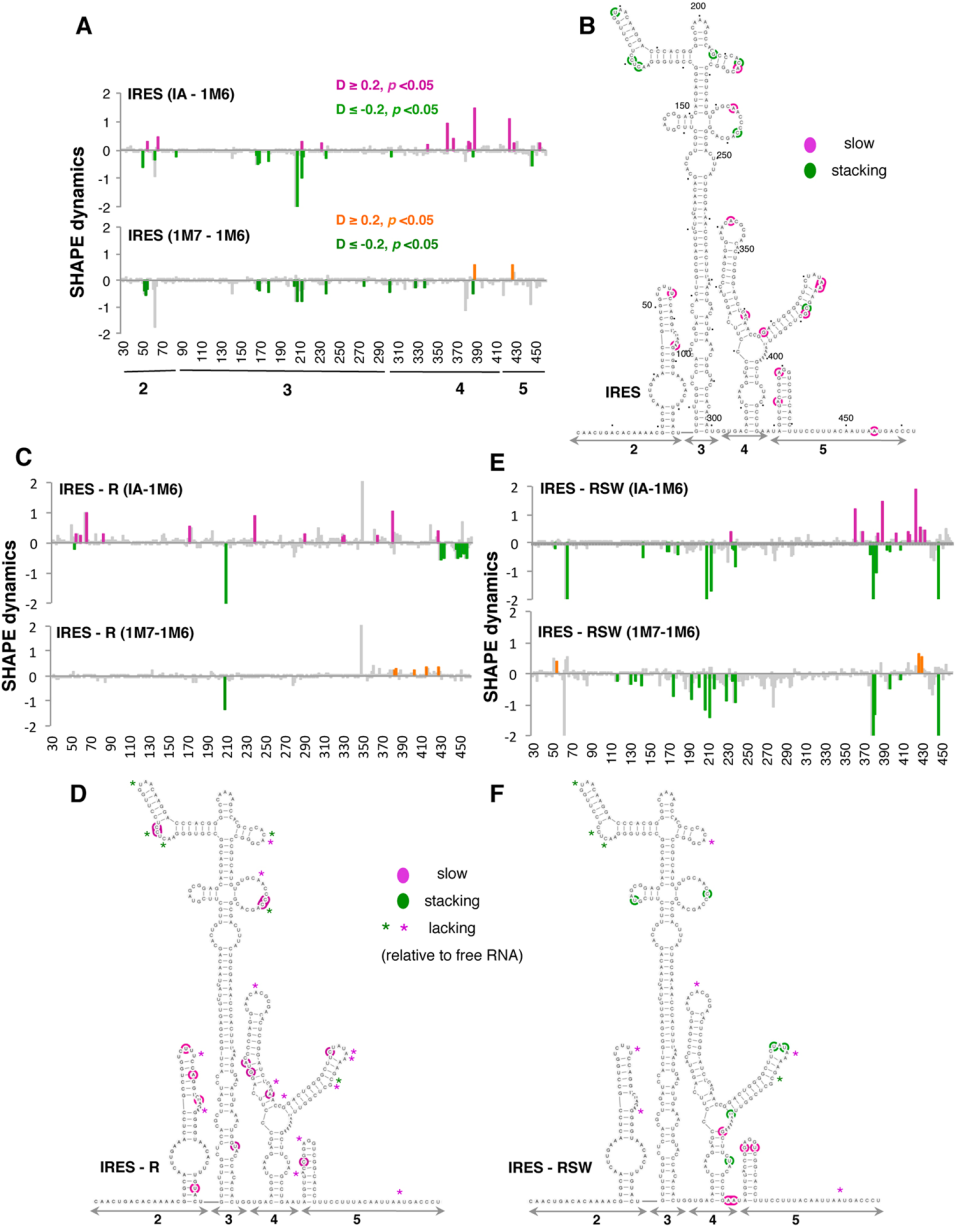
RNA dynamics of the IRES upon incubation with ribosome fractions. Differential SHAPE reactivity as a function of the nucleotide position depicting IA-1M6 and 1M7-1M6 statistically significant differences obtained for the free RNA (**A**). The secondary structure of the IRES shows IA enhanced reactivity (slow, pink) and 1M6 enhanced reactivity (stacking, green) (**B**). Nucleotides are numbered every 50 positions, dots mark every 10 positions. (**C**,**E**) Differential SHAPE reactivity for IRES-R complexes, or IRES-RSW complexes, and (**D**,**F**) changes in RNA dynamics for IRES-R, or IRES-RSW. Asterisks depict positions lacking differential reactivity relative to the free RNA.

Reagent-specific differential reactivity obtained with the IA-1M6 difference for the free RNA is shown in Fig. 7A. Specifically, statistically significant increased reactivity towards IA was noticed in nucleotides mostly located on domains 4 and 5 (pink bars). Conversely, residues mostly located on domain 3 displayed enhanced reactivity toward 1M6 (green bars). Next, to identify nucleotides potentially involved in tertiary interactions, we obtained the SHAPE reactivity profile with an additional fast-reacting compound 1M7 (Dataset 3), which unlike 1M6 does not stack with nucleobases^7^. The statistically significant reactivity differences 1M7-1M6 (Fig. 7A) revealed that positions 168, 170, 179, 208, 213, 238, and 388 were specific 1M6 enhancements, reflecting reagent stacking at accessible nucleobases^4^. Hence, they are candidates to be involved in long-range interactions. Representation of the differentially reagent-specific reactive nucleotides on the secondary structure model showed that nucleotides with slow folding dynamics were mainly located in domains 4 and 5 while those likely involved in long-range interactions were mainly found in the apical region of domain 3 (Fig. 7B).

The ligand-bound differential nucleotides obtained for IRES-R complexes denoted slow folding positions spread on all domains, while residues that increased their reactivity toward 1M6 were located in domain 5 (Fig. 7C). To identify nucleotides involved in stacking (1M6 specific) we calculated the difference 1M7-1M6 for the IRES-R complexes (Fig. 7C). We noticed that the statistically significant IA-1M6 differences present in domain 5 were not detected in the 1M7-1M6 profiles; hence, these positions were discarded as candidates for long-range interactions. Representation of the statistically significant differences on the secondary structure of the IRES revealed that nucleotides undergoing slow motions observed in the IRES-R complexes relative to the free RNA are located in the same domains (2, 3 and 4), but on different positions (pink residues in Fig. 7D). In addition, the stacking nucleotides within domain 3 observed in the free RNA were not detected in the presence of the R extract (asterisks in Fig. 7D), with the exception of nt 208. Therefore, the presence of native ribosomes alters significantly the RNA dynamics pattern, affecting in particular the apical region of domain 3.

In contrast to the IRES-R complexes, the RNA dynamics pattern of IRES-RSW complexes (Fig. 7E) resembles the free RNA (Fig. 7A). In addition, representation of the differential IA-1M6 nucleotides (taking into account only 1M6 specific positions according to 1M7-1M6 differences) revealed that, relative to the free RNA, changes were concentrated on the apical region of domain 3 and domain 4 (green residues in Fig. 7F). Thus, we conclude that, relative to free RNA, global RNA dynamics changes are observed in the IRES-R complexes (Fig. 7D), acquiring slow dynamics. In contrast, the RNA dynamics of IRES-RSW complexes resembles the free RNA (Fig. 7F), affecting locally the IRES region.

## Discussion

Here, we have investigated the conformational flexibility and the RNA dynamics of a model IRES element taking advantage of differential SHAPE methodology. Differential nucleotides comprise non-canonical and tertiary RNA structures highlighting residues that adopt specific structural features on different timescales^4^. Our data show that, on the free RNA, nucleotides reaching the final conformation on long timescales are placed on domains 4–5 of the IRES region upstream of the start codon. In contrast, nucleotides candidate to be involved in tertiary interactions are mostly located on the apical region of domain 3, consistent with the finding that mutations disrupting the native structural organization of this region impaired IRES activity^23,24^.

**Figure 8:**
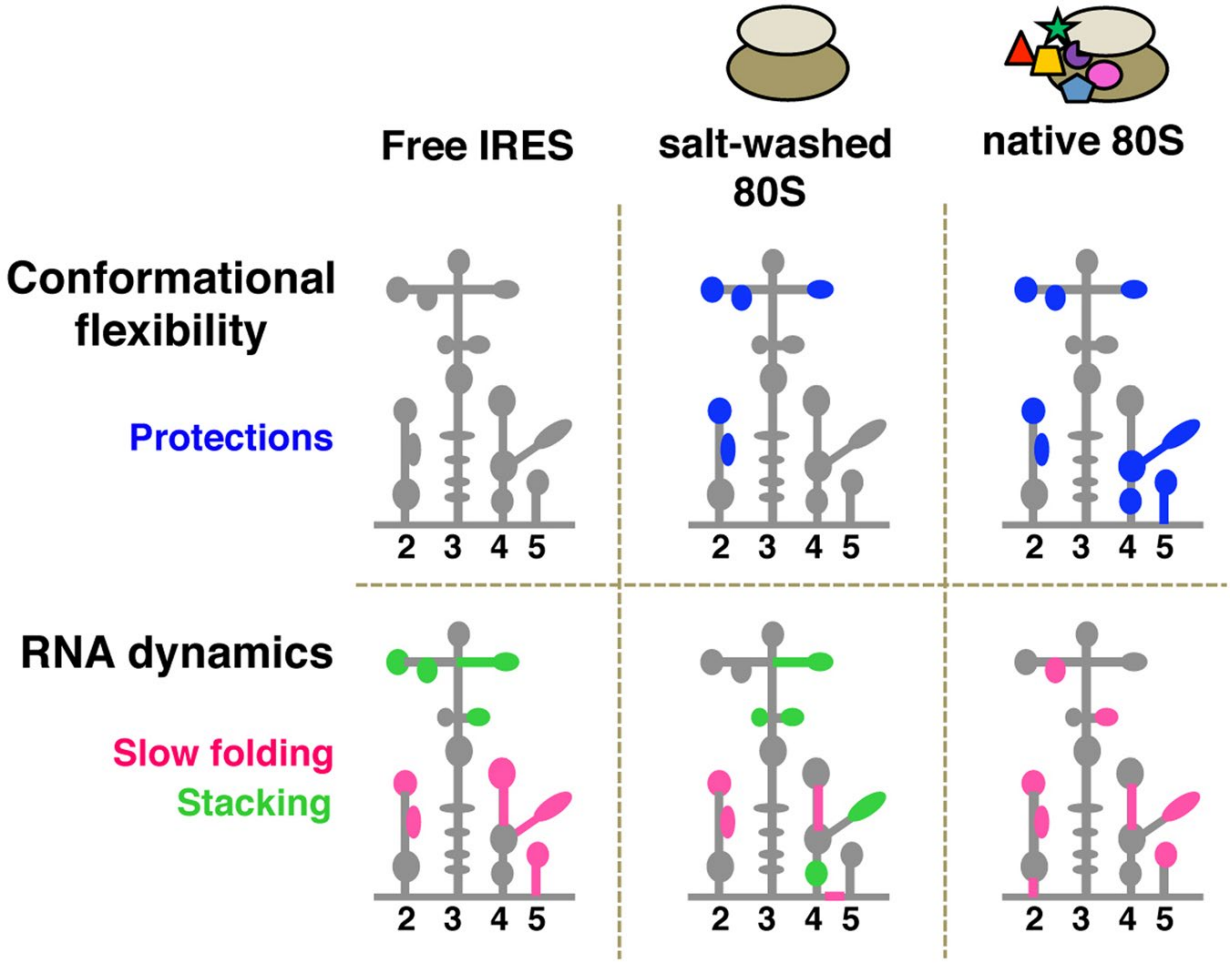
Schematic representation of the changes in conformational flexibility and RNA dynamics of the IRES in the presence of the indicated ribosome fractions relative to free RNA.

Given that local RNA structure could be pre-formed to facilitate the interaction with ligands^25^, we decided to study the IRES conformational flexibility in the presence of various ribosomal fractions. The results obtained illustrated two structural features of the IRES region. First, ribosomes free of factors (RSW) modified the conformational flexibility of domains 2 and 3 of the IRES element (the 5´end domains) (Fig. 8). Second, native ribosomes (R) induced additional structural changes within domains 4 and 5. Moreover, supplementing RSW with the F fraction restored the RNA conformation of the IRES incubated with native ribosomes (Fig. 4). Taken together, these results lead us to suggest that there is a division of functions among the modular domains of the IRES. Hence, according to the conformational flexibility changes observed by differential SHAPE, we hypothesize that the individual domains of the FMDV IRES contain separate sites for ribosome-interaction and eIFs-binding.

It is known that ligand binding modifies the conformation of RNA molecules altering their energy landscape and guiding RNA dynamics^2^. Our study reveals that IRES-R and IRES-RSW complexes affect differentially the RNA dynamics of the IRES region. Thus, the ligand-free RNA resembles the RSW RNA dynamics (Fig. 7A,C), with small differences that point to stacking residues in subdomain K of domain 4 (green marks in Fig. 8). In contrast, the R fraction induced global changes on the RNA dynamics of the IRES. The slow differential positions noticed in the free RNA were absent in the IRES-R complexes, and slow folding nucleotides were detected in different positions of the IRES domains (Fig. 7B). Moreover, nucleotides 170 and 238, which showed 1M6-enhanced reactivity in the free RNA switch to IA-enhanced reactivity in the presence of native ribosomes (Fig. 8, pink marks). This is in part consistent with earlier experimental data and RNA modeling that proposed the existence of a tertiary interaction affecting the GNRA motif and the 170 bulge with the C-rich loop^26–28^. In addition, stacking nucleotides were not observed in the presence of the R fraction, with the exception of position 208, suggesting that the conformation of this residue was independent of ligand binding. Taken together, ribosome-associated factors modulate the global dynamic folding of the IRES.

In spite of the fact that earlier work reported the need of eIFs for the *in vitro* assembly of 48S complexes with picornavirus IRESs^29^, direct interactions between the 40S ribosomal subunit and the related EMCV IRES have been recently described^30^. In agreement with these studies, our results showed that both ribosomal subunits induced conformational changes on domains 2 and 3 of the FMDV IRES (Fig. 5B,C), but also that factors were required to achieve the IRES final conformation. In fact, the domains involved in 40S recruitment in EMCV IRES (domains H and I) are homologous of FMDV IRES domains 2 and 3 (this work). Nucleotides on FMDV domain 2 and EMCV domain H are coincident; however, differences on domain 3 might be due to the use of different SHAPE reagents.

The observation that the dissociated 40S and 60S ribosomal subunits could interact with the IRES region *in vitro* led us to analyze whether the IRES-containing RNA could remain bound to the ribosomal subunits, lacking translation factors, in whole cell lysates. These results indicated that the ratio of IRES/cap RNA bound to ribosomal subunits was higher for the IRES-RNA (Fig. 6C). We interpret these results as a trait of the IRES-RNA to remain preferentially associated to ribosomal subunits relative to capped RNAs. These data illustrate how RNA structure-based control determines RNA function.

Base pairing between the mRNA and the 18S ribosomal RNA has been suggested as a mechanism used by viral IRES elements to recruit the 40S subunit^31,32^. Potential base pairs between the FMDV IRES and the 18S RNA positions 815–1193 were predicted using IntaRNA^33^ (Table 1). In particular, the sequences within domain 2 sharing complementarity with 18S positions were also predicted to interact with the HCV IRES^34^. For 28S RNA, a higher number of complementary sequences were identified within domain 2 (Table 1), consistent with the observation that intensity of protections induced in domain 2 by the 60S subunits were slightly stronger than those induced by the 40S subunits (Fig. 5C). In agreement with our data, interaction of viral RNA structural motifs with 60S subunits has been previously reported^35,36^.

**Table 1:** Predicted base pairs for domain 2 of the FMDV IRES with 18S and 28S rRNA*.

Domain (nt position)	IRES sequence (5′-3′)	rRNA sequence (5′-3′)	nt position	rRNA
D2 37–40	UGAA	UUCG	939–942	18S h23
D2 50–54	GGUCU	GGACC	985–989	18S h23
D2 60–68	GGUCUAG**A**GG	UU**C**UUGGACC	965–974	18S h23
D2 36–42	UUGAAAC	GUUUUAA	4357–4362	28S h88
D2 50–72	GGUCUUUC**CA**GGUC**U**AGAGGGGU	GCCUCUCCAG**U**CC**GCC**GAGGGCGCACC	1436–1461	28S h30ES9
D2 51–56	GUCUUU	GGAGGC	3970–3975	28S h77
D2 51–57	GUCUUUC	GAAAGAU	1523–1528	28S h32
D2 51–57	GUCUUUC	GAGAGAU	2448–2454	28S h52
D2 51–57	GUCUUUC	GAAGGGC	4272–4277	28S h85
D2 51–57	GUCUUUC	GAGAGGC	4908–4914	28S h98ES39b
D2 52–58	UCUUUCC	GGAAAGA	3899–3905	28S h73
D2 67–84	GAGGGGU**AAC**ACUUUGUAC	GUGCGGAGUGCCCUUCG	4868–4883	28S h98ES39
D2 68–72	AGGGGU	GCCUCU	4695–4700	28S h97
D2 74–80	ACACUUU	GAGGUGU	3953–3959	28S h76

^*^Prediction of base pairs was performed using IntaRNA. Only ribosomal RNA nucleotides located in flexible regions (according to^54^) were considered. Unpaired nts are indicated in bold.

In summary, our work suggests the presence of short motifs within the IRES with the capacity to interact with the ribosomal particles. Since all FMDV IRES domains are necessary but not sufficient to promote internal initiation^19^, our findings support the idea that RNA motifs present in domains 2 and 3 could define a functional building block, as shown for other RNAs^37^. We hypothesize that IRES elements could be derived from the association of distinct building blocks containing RNA motifs with specific features. For instance, RNA motifs able to contact the ribosomal subunits, joined to RNA structural motifs providing the interaction with eIFs and RNA-binding proteins. Individually, none of them contain full IRES activity, consistent with the observation that viral IRES elements function as single entities^38,39^. These RNA building blocks could be useful to design synthetic IRESs with novel functional features.

## Methods

### Constructs

The construct expressing the monocistronic IRES RNA was previously described^40^. RNA was synthesized *in vitro* using a plasmid linearized with SphI. Transcription was performed using T7 RNA polymerase, as described^41^. Synthesis of full-length products was verified by denaturing gel electrophoresis. The plasmid pIRES-luc was generated substituting the EcoRI-BamHI fragment of Tagged-IRES construct (Addgene plasmid # 35570^42^) by the EcoRI-BamHI fragment of pGEM-IRES^43^. The plasmid pCAP-luc was generated in two steps. First, the BamHI site was substituted by EcoRI site in plasmid Tagged-IRES using the QuikChange mutagenesis procedure (Agilent Technologies) with the primers 5′-TTTTTGGCGTCTTCCATGAATTCTCGAGCTCAGGGTCATT and 5′-AATGACCCTGAGCTCGAGAATTCATGGAAGACGCCAAAAA. Then, ligation of the EcoR1 digested plasmid generated the pCAP-luc construct. The sequences were verified by DNA sequencing (Macrogen).

### Differential SHAPE reactivity reactions

Prior to treatment, *in vitro* synthesized RNA was folded by heating at 95 °C for 2 min, snap cooling on ice for 2 min, and subsequently incubated in a final volume of 18 μl of folding mix (100 mM HEPES pH 8.0, 100 mM NaCl, 5.25 mM MgCl_2_) for 20 min at 37 °C^40^. Prefolded RNA (2 pmol) was incubated with 6.5 mM 1-methyl-7-nitroisatoic anhydride (1M7), 1-methyl-6-nitroisatoic anhydride (1M6) or isatoic anhydride (IA) (Thermo-Fisher) for 70 s, 3 min or 36 min, respectively^4,6,44^ at 37 °C. Untreated RNA was incubated with DMSO. Treated and untreated RNAs were precipitated and finally resuspended in 10 μl of 0.5x TE.

For differential SHAPE probing of RNA-ribosome, RNA-40S or RNA-60S subunits, complexes were assembled in folding buffer in the presence of 4-fold ribosome^30^ or ribosomal subunits during 10 min at 37 °C. RNA-S100 complexes were assembled using 1 µg of total protein. Then, RNA alone or incubated with the indicated fractions was treated with IA, 1M6, or 1M7.

### Primer Extension reactions

Treated and untreated RNA (2 pmol) was incubated with the fluorescent primer 5′-NED-TAGCCTTATGCAGTTGCTCTCC (0.1 µM) at 65 °C for 5 min, then at 35 °C 5 min, and 4 °C 1 min. Primer extension reactions were conducted in a final volume of 16 μl containing reverse transcriptase (RT) buffer (50 mM Tris–HCl pH 8.3, 75 mM KCl, 3 mM MgCl_2_, 7.5 mM DTT), 10 U RNase OUT (Thermo-Fisher), 1 mM each dNTP and 60 U of Superscript III RT (Thermo-Fisher). Reverse transcriptase reactions were performed during 30 min at 52 °C, followed by 15 min at 70 °C. Primer extension products were resolved by capillary electrophoresis. The 5′-FAM-TAGCCTTATGCAGTTGCTCTCC primer was used for the sequencing ladder, using 2.5 pmol of untreated RNA in the presence of 0.1 mM ddCTP, 30 min at 52 °C RT reaction^45^.

### SHAPE reactivity data analysis

SHAPE electropherograms were analyzed using QuSHAPE software^46^. The reactivity values obtained for each untreated RNA (DMSO) were subtracted from the corresponding (IA, 1M6, or 1M7) treated RNA to obtain the net reactivity for each nucleotide for the free RNA, or RNA incubated with S100 fraction, native ribosomes (R), salt-wash ribosomes (RSW), factors disassociated from the native ribosomes (F), or the ribosomal subunits (40S) and (60S). Quantitative SHAPE reactivity for individual datasets were normalized to a scale spanning 0 to 2 in which 0 indicates an unreactive nucleotide and the average intensity at highly reactive nucleotides is set to 1. Data from at least 3 independent assays were used to calculate the mean (±SD) SHAPE reactivity [see Dataset 1 (IA), Dataset 2 (1M6) and Dataset 3 (1M7)]. For footprint analysis of ribosomal extracts on IRES RNA, the normalized mean SHAPE reactivity obtained for the free RNA was subtracted to the mean reactivity obtained for each extract.

For differential SHAPE analysis, the normalized mean 1M6 reactivity was subtracted to IA or 1M7 mean reactivity. The statistical significance of the SHAPE reactivity data obtained under different conditions from at least 3 independent experiments was determined by the unpaired two-tail Student´s *t*-test. Only nucleotide positions with absolute difference (D) ≥ 0.2 arbitrary units and *p* value < 0.05 were considered statistically significant. RNA secondary structures were visualized with VARNA.

### Cell culture and subcellular fractionation

HEK293 cells were maintained in standard conditions in Dulbecco’s modified Eagle’s medium (DMEM). The S30, S100, R and RSW fractions were obtained as described^47^ with little modifications. Briefly, HEK293 cells grown to 90% confluence were lysed in buffer 1 (15 mM Tris-HCl pH 7.4, 80 mM KCl, 5 mM MgCl_2_, 1% Triton-X-100, protease inhibitors (Roche)). Cell debris was discarded by spinning at 14000 g 10 min 4 °C, twice. The supernatant of the second spinning is the S30 fraction. S30 centrifugation at 95000 rpm during 1.5 h using the TLA100.3 rotor yielded the S100 fraction (supernatant), and the ribosomes plus associated factors (pellet). The ribosomes pellet was resuspended in buffer 3 (10 mM HEPES pH 7, 50 mM KCl, 10 mM MgCl_2_, 5 mM β-ME) to yield the R fraction. To prepare the fraction containing ribosomes free of associated factors, the ribosomal pellet was dissolved in high-salt buffer 2 (15 mM Tris-HCl pH 7.4, 500 mM KCl, 5 mM MgCl_2_, 2 mM DTT, 290 mM sucrose), loaded in a discontinuous sucrose gradient, centrifuged at 4 °C 95000 rpm 2 h using a TLA100.3 rotor. The pure ribosomes pellet (RSW) was resuspended in buffer 3. The supernatant of the ultracentrifugation was dialyzed against to buffer 3 to prepare the F fraction containing the factors that disassociated from the ribosomes. The total protein content in S30, S100, and F fractions was measured by the Bradford assay; the ribosome concentration in R and RSW fractions was determined as 14 units A260 = 1 mg/ml.

### Immunodetection

Proteins from cell lysates were separated by SDS-PAGE and probed with the indicated antibodies. Commercial antibodies were used to detect eIF4E (Transduction laboratories), RACK1, eIF2α, eIF4G (Santa Cruz), eEF2 (Cell Signaling), eIF4B, and Gemin5 (Novus). The ribosomal proteins P0, P1 and P2 were detected with the monoclonal antibody 3BH5^48^. Appropriate secondary antibodies (Thermo-Fisher) were used according to the manufacturer instructions. Protein signals were visualized with ECL plus (Millipore). Quantification of the signal detected was done in the linear range of the antibodies.

### Purification of 40S and 60S ribosomal subunits

Ribosome dissociation profiles were prepared from HEK293 cells, as described^47^. A western blot analysis of the 40S and 60S purified subunits is shown in Supplementary Fig. S6. The concentration of 40S or 60S subunits  was calculated as 1 A260 unit = 53 pmol/ml for 40S and 32 pmol/ml for 60S^22^.

### Transfections and ribosome dissociation profiles

HEK293 monolayers (about 70% confluent) were transfected with the plasmid pCAP-luc or pIRES-luc using lipofectamine (Thermo-Fisher). Cell lysates were prepared 24 hpt in lysis buffer (50 mM Tris-HCl pH 7.8, 100 mM NaCl, 0.5% NP40) for determination of luciferase activity, or in buffer B for RNA-ribosomal subunits association assays, as described^47^.

### Luciferase activity assays

IRES or CAP activity was quantified as the expression of luciferase normalized to the amount of protein (determined by Bradford assay) in the lysate obtained from transfected HEK293 cell monolayers. Each experiment was repeated independently at least three times. Values represent the mean ± SD.

### Monitoring RNA-ribosomal subunits association by RTqPCR

Total RNA was isolated from transfected cell lysates and from dissociation gradient fractions corresponding to 40S and 60S peaks using TRIzol reagent (Thermo-Fisher). Equal amount of RNAs were used to synthesize cDNA using SuperScript III (Thermo-Fisher) and hexanucleotide mix (Merck) as primer for the reverse transcription reaction. Primers for Quantitative polymerase chain reaction (qPCR) were designed (Primer3 software, http://bioinfo.ut.ee/primer3-0.4.0/primer3/) and tested for amplification efficiency. qPCR was carried out with GoTaq qPCR Master Mix (Promega) according to the manufacturer’s instructions on an ABI PRISM 7900HT Fast Real-time PCR system (AppliedBiosystems) using primers specific to Luciferase mRNA (5′-TGGCAGAAGCTATGAAACGA and 5′-ATAAATAACGCGCCCAACAC). Values were normalized against an endogenous control mRNA RPL11 (5′-GTGCTGGGTAGGCCAG and 5′- TTCTGCTGGAACCAGCG). The comparative cycle threshold (CT) method^49^ was used to quantify the results.

### *In silico* prediction of RNA-RNA long-range interactions

IntaRNA^33^ was used to predict RNA-RNA long-range base pairings incorporating the accessibility of target sites under default parameters (minimum seed size 7, mismatches 0). This approach computes a combined energy score of the interaction as the sum of the free energy of hybridization and the free energy required for making the interaction sites accessible.

### 3D structure modeling

The RNA secondary structure of the IRES element under different conditions was predicted using RNAstructure^50^ imposing SHAPE reactivity values as pseudo-free energy change constraints together with nearest neighbor thermodynamic parameters using -0.8 kcal/mol and 2.6 for the intercept (b) and slope (m), respectively^51^. These secondary structures were used as input in RNAComposer^52^ to model the 3D RNA structure using default parameters. 3D structures graphics were performed with the UCSF Chimera package^53^.

## Electronic supplementary material

Supplementary Information

Dataset1

Dataset 2

Dataset 3
