# Supplementary material for: Ribosome-dependent conformational flexibility changes and RNA dynamics of IRES domains revealed by differential SHAPE
Source: Sci Rep. 2018 Apr 3;8:5545. doi: 10.1038/s41598-018-23845-x (PMC5882922; doi:10.1038/s41598-018-23845-x)
Supplement: Supplementary file 1 — Supplementary Information [file 41598_2018_23845_MOESM1_ESM.pdf]

SUPPLEMENTARY INFORMATION

Ribosome-dependent conformational flexibility changes and RNA dynamics of IRES domains revealed by differential SHAPE

Gloria Lozano[¶], Rosario Francisco-Velilla[¶], and Encarnacion Martinez-Salas*

Centro de Biología Molecular Severo Ochoa, Consejo Superior de Investigaciones Científicas - Universidad Autónoma de Madrid, Nicolás Cabrera 1, 28049 Madrid, Spain

[¶]These authors contributed equally to the paper as first authors.

SUPPLEMENTARY FIGURES

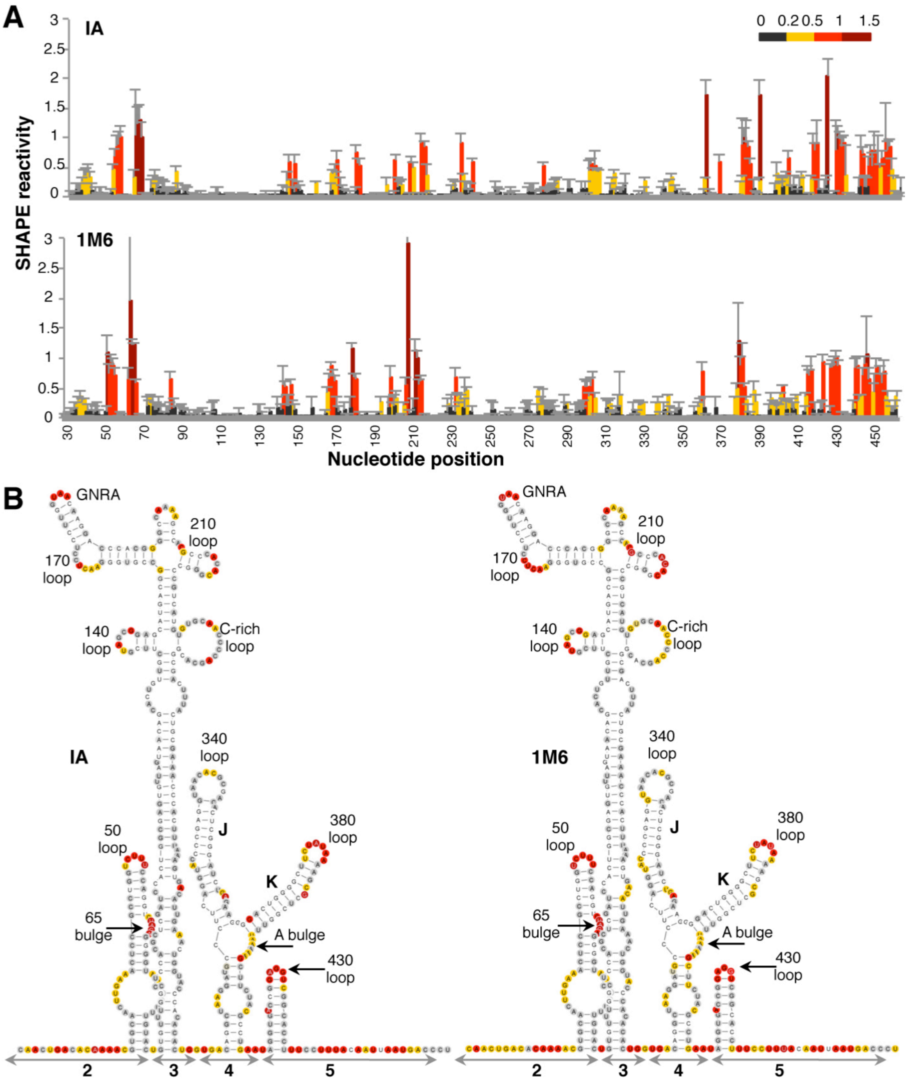

Supplementary Figure S1. **Local flexibility of the IRES determined with IA or 1M6 SHAPE reagents**. **A**. Normalized SHAPE reactivity towards IA (n=9) or 1M6 (n=5) as a function of the nucleotide position. Values correspond to the mean ± SD. Normalized RNA reactivity is colored according to the scale shown on the right. **B**. RNA secondary structure models showing IA or 1M6 reactive positions.

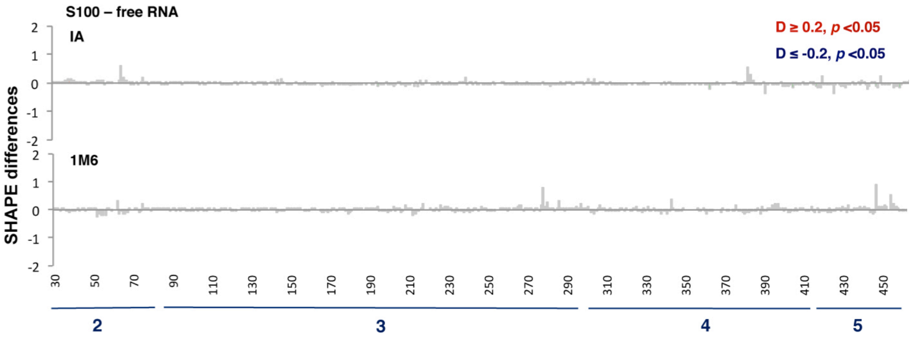

Supplementary Figure S2. Conformational changes in the local flexibility of the IRES incubated with S100 cellular fraction. Grey bars depict SHAPE differences with *p*-values >0.05 and/or absolute differences <0.2.

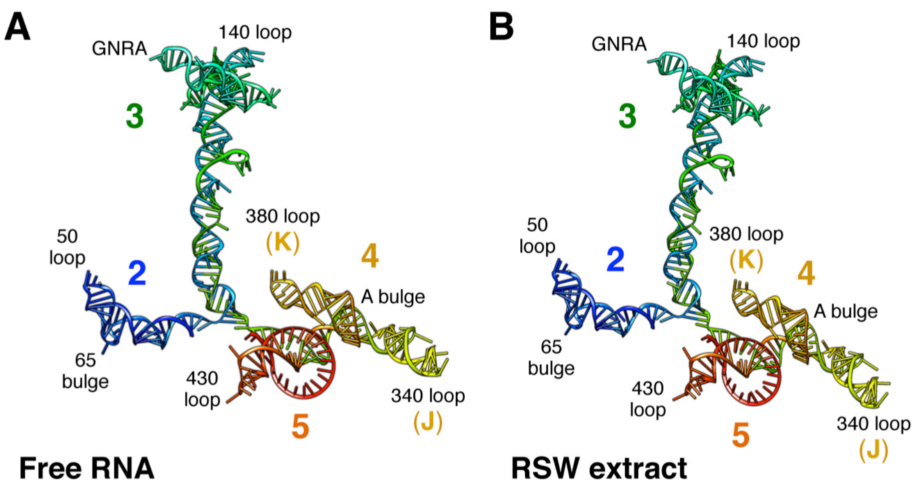

Supplementary Figure S3. Predicted 3D structure models for the IRES imposing SHAPE reactivity values obtained for the free RNA (**A**) and upon incubation of the IRES transcript with the RSW extract (**B**). Domains 2, 3, 4, and 5, subdomains J and K of domain 4, as well as the GNRA tetraloop, loops, and bulges referred to as in the text are indicated.

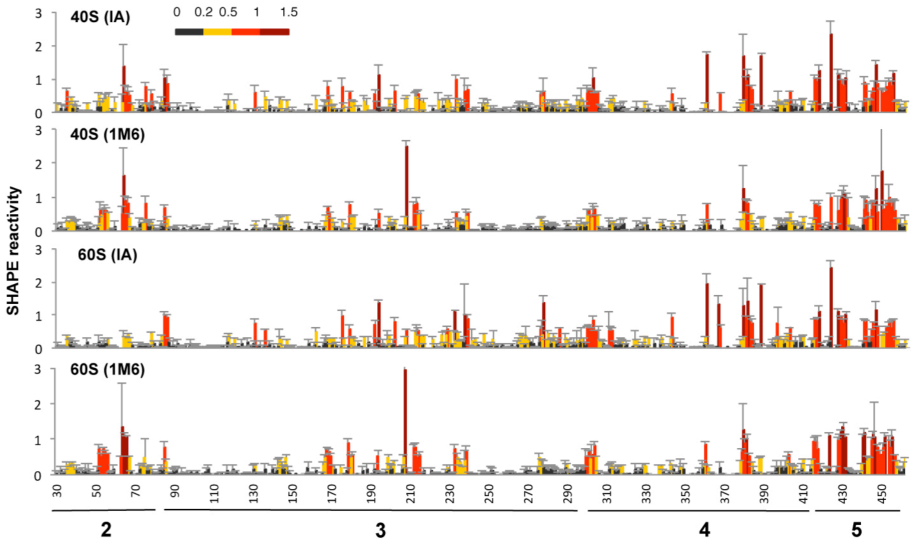

Supplementary Figure S4. Local flexibility of the IRES incubated with ribosomal subunits. Normalized SHAPE reactivity towards IA or 1M6 as a function of the nucleotide position incubated with 40S or 60S subunits. RNA reactivity is colored according to the scale shown on the left. Values correspond to the mean ± SD of at least three independent experiments.

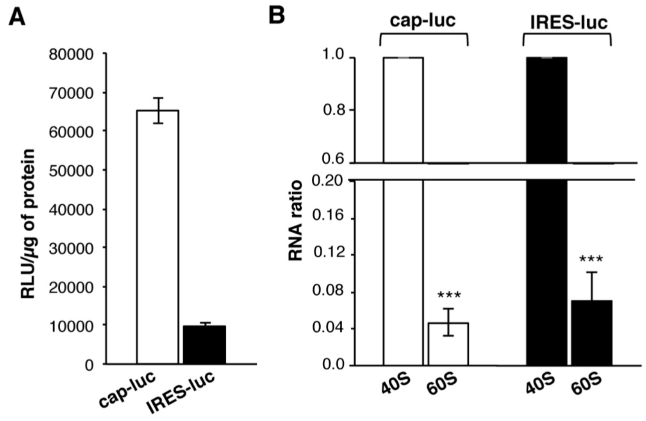

Supplementary Figure S5. Association of cap-RNA and IRES-RNA to ribosomal subunits. **A**. Cap- and IRES-dependent translation initiation was monitored as luciferase activity. White and black bars depict cap- and IRES-dependent translation, respectively. **B**. Analysis of cap-RNA and IRES-RNA levels associated to 60S subunits relative to 40S subunits. White and black bars depict cap-luc and IRES-luc RNA copies, respectively. Values represent the mean ± SD obtained in two independent assays (***P < 0.001 by Student´s t test).

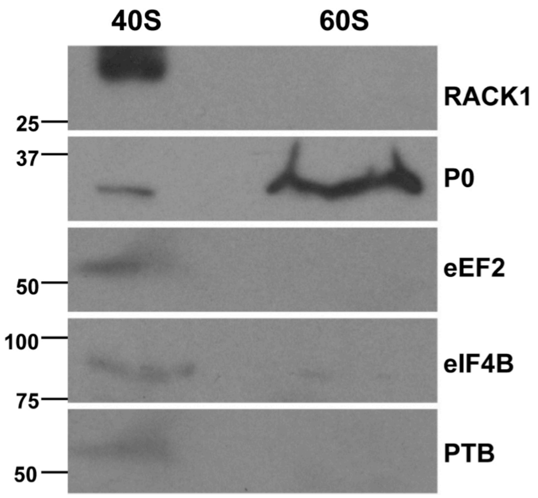

Supplementary Figure S6. Preparation of 40S and 60S ribosomal subunits. HEK293 ribosomal subunits were prepared from high-salt treated sucrose gradients, as described[45]. The presence of expected 40S and 60S ribosomal proteins (RACK1 and P0, respectively) was analyzed by Western blot on the same membrane. The initiation factor eIF4B and the IRES-interacting protein PTB were analyzed as a control of the preparation. This figure shows horizontal slices of the WB carried out for each factor. Images of the un-cropped WB for each factor are shown in Supplementary Fig. S8).

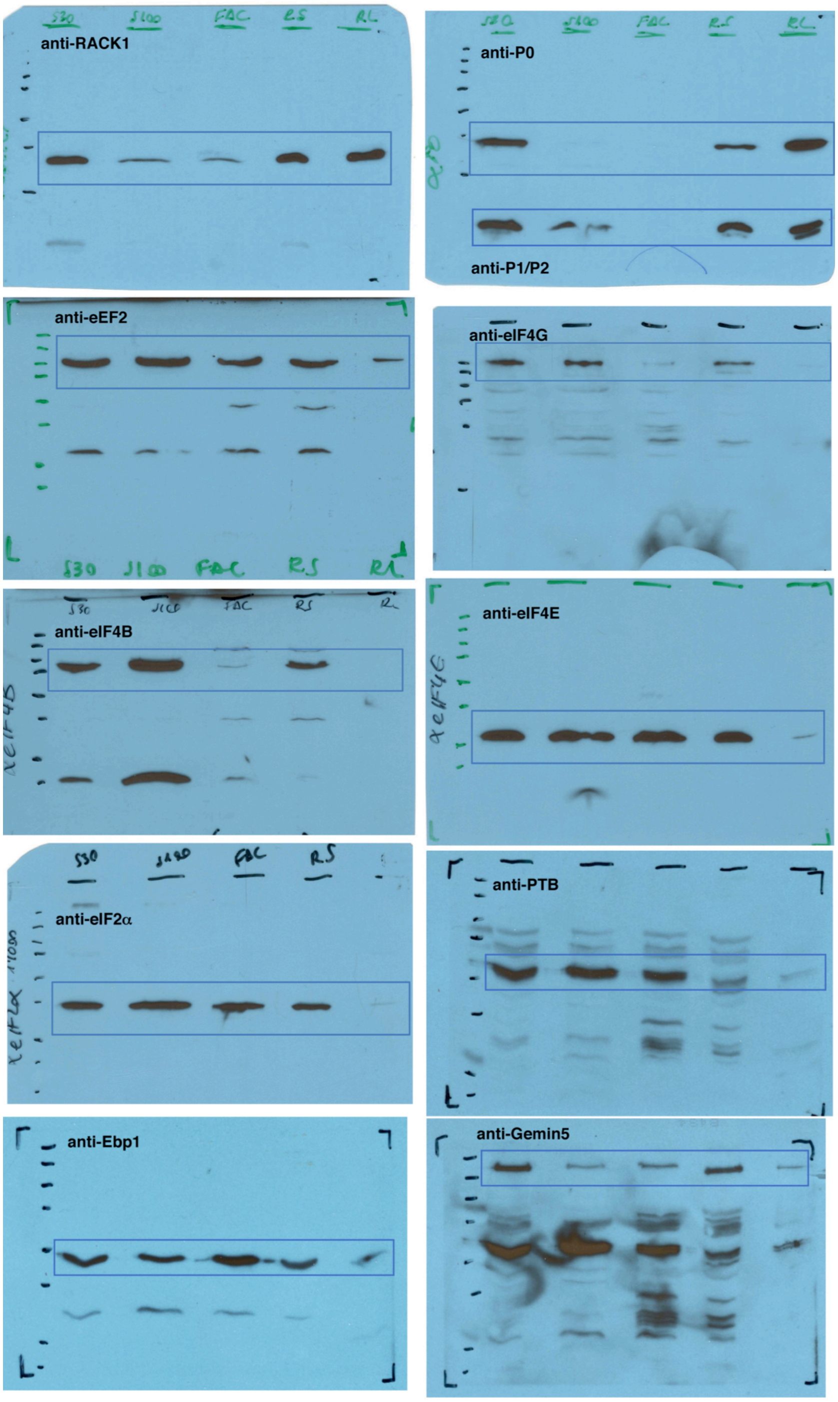

Supplementary Figure S7. Images of un-cropped Western blots conducted for each protein (corresponding to Fig. 2). The 3BH5 antibody recognizes the C-terminal domain of P0, P1 and P2, conserved in all organisms.

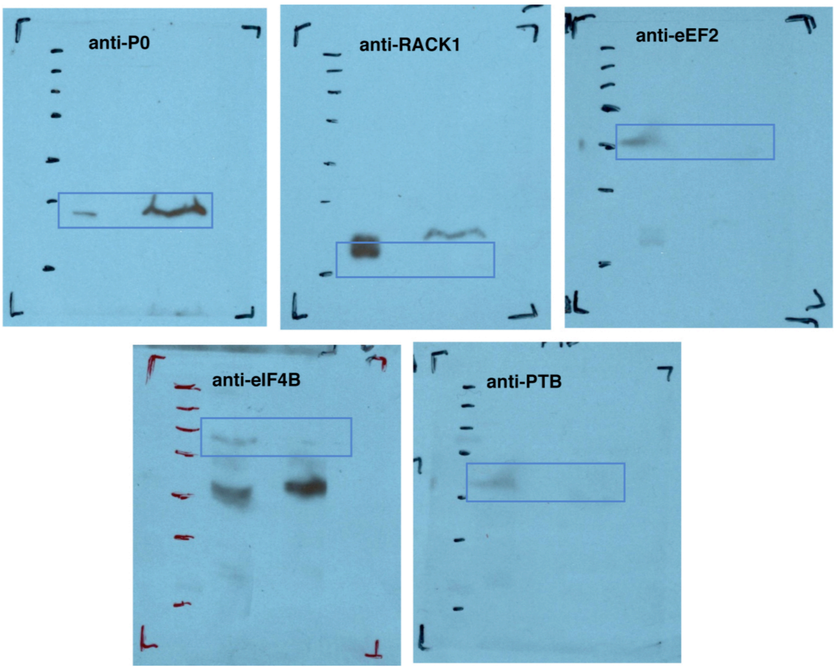

Supplementary Figure S8. Images of un-cropped Western blots conducted for the indicated proteins (corresponding to Supplementary Fig. S6). The secondary antibody used to detect RACK1 also recognizes the ribosomal protein P0.
